# Spiritual Well-Being, Perceived Social Support, and Death Anxiety Among Primigravid Women: A Cross-Sectional Study

**DOI:** 10.3390/healthcare14183046

**Published:** 2026-09-17

**Authors:** Ezgi Şahin, Cennet Çiriş Yildiz

**Affiliations:** 1 Department of Midwifery, Faculty of Health Sciences, Giresun University, Giresun 28200, Türkiye; ezgi.sahin@giresun.edu.tr; 2 Department of Administration in Nursing, Faculty of Health Sciences, Istanbul Aydin University, Istanbul 34295, Türkiye

**Keywords:** primigravid women, pregnancy, spiritual well-being, death anxiety, perceived social support, antenatal care

## Abstract

**Highlights:**

**What are the main findings?**

- Higher spiritual well-being and perceived social support were associated with lower death anxiety among primigravid women.
- An exploratory analysis identified a significant cross-sectional indirect association between spiritual well-being and death anxiety through perceived social support.

**What are the implications of the main findings?**

- Spiritual well-being and social support may be relevant psychosocial resources when assessing death anxiety during a first pregnancy.
- The findings provide exploratory evidence that spiritual well-being and perceived social support may be relevant when considering psychosocial needs in antenatal care.

**Abstract:**

**Background/Objectives:** Pregnancy may involve psychological and existential concerns, particularly for women experiencing pregnancy for the first time. This study examined the relationships among spiritual well-being, perceived social support, and death anxiety in primigravid women and explored the cross-sectional indirect association through perceived social support. **Methods:** This cross-sectional study included 390 primigravid women attending an antenatal outpatient clinic in northern Türkiye. Data were collected using the Three-Factor Spiritual Well-Being Scale, Multidimensional Scale of Perceived Social Support, and Death Anxiety Scale. Pearson correlation, hierarchical multiple regression, and an exploratory cross-sectional indirect association analysis using PROCESS Model 4 with 5000 bootstrap samples were performed. **Results:** Spiritual well-being was positively correlated with perceived social support (r = 0.495, *p* < 0.001) and negatively correlated with death anxiety (r = −0.554, *p* < 0.001). Perceived social support was also negatively correlated with death anxiety (r = −0.522, *p* < 0.001). After adjustment for the prespecified covariates, spiritual well-being (β = −0.152, *p* < 0.001) and perceived social support (β = −0.145, *p* < 0.001) remained negatively associated with death anxiety. A statistically significant adjusted indirect association was observed in the specified cross-sectional model (B = −0.91, 95% percentile bootstrap CI [−1.44, −0.44]). **Conclusions:** Higher spiritual well-being and perceived social support were associated with lower death anxiety among primigravid women. A statistically significant adjusted cross-sectional indirect association between spiritual well-being and death anxiety through perceived social support was also observed; however, this finding does not establish mediation, temporal ordering, or causality.

## 1. Introduction

Pregnancy is a major life transition involving simultaneous biological, psychological, and social changes. For women experiencing pregnancy for the first time, adapting to these changes may be particularly challenging because of unfamiliarity with the process, uncertainty about pregnancy and childbirth, and adjustment to a new maternal role [1,2]. Anxiety and psychological distress are common during the antenatal period and may have consequences for both maternal well-being and pregnancy outcomes [3,4,5]. Pregnancy should therefore be considered not only as a physiological process but also as a period in which psychological adjustment and broader existential concerns may be relevant [1,3].

Death anxiety refers to cognitive and emotional responses arising from awareness of one’s own mortality [6,7]. It is conceptually distinct from pregnancy-related anxiety, which encompasses pregnancy-specific concerns such as fetal health, maternal well-being, bodily changes, and the course of pregnancy [8], and from fear of childbirth, which focuses more specifically on anticipated labor and birth [9]. Although concerns about maternal or fetal complications may overlap with these forms of pregnancy-related distress, they do not necessarily constitute death anxiety unless they specifically involve death or mortality [9,10]. Death anxiety is a broader existential construct that is not limited to a particular health condition or life event and has been associated with anxiety, depression, and other forms of psychological distress [6,7]. The Death Anxiety Scale used in this study assesses general thoughts and emotions concerning mortality, including uncertainty about death, thinking about or witnessing death, and anticipated suffering [11]. As it is not a pregnancy-specific measure, the scores cannot be interpreted as indicating that death anxiety originates from pregnancy. Nevertheless, examining this broader construct among primigravid women may provide insight into an underexplored aspect of their psychological well-being. However, because the present study did not include multigravid or non-pregnant comparison groups, it cannot determine whether death anxiety is heightened by or attributable to a first pregnancy.

Spiritual well-being may be one of the personal resources involved in coping with such concerns. It is a multidimensional concept related to finding meaning in life, maintaining a sense of inner coherence, and dealing with existential uncertainty [12]. Higher spiritual well-being has been associated with lower anxiety and psychological distress and better mental well-being [12,13]. Similar findings have been reported during pregnancy. Studies among pregnant women suggest that spiritual well-being is positively associated with psychological resilience and life satisfaction and may contribute to adjustment during pregnancy [14,15]. These findings suggest that spiritual well-being may be particularly relevant when women face uncertainty and concerns that extend beyond the physical aspects of pregnancy.

Psychological adjustment during pregnancy is also closely related to the social resources available to women. Perceived social support reflects an individual’s perception of the emotional, informational, and practical support available from partners, family members, friends, and others in their social environment. Greater perceived social support during pregnancy has been associated with lower levels of anxiety, stress, and depression [3,16]. Social support may also buffer the psychological burden of stressful experiences by strengthening coping resources and providing a greater sense of security [12].

The conceptual framework of this study was informed by meaning-making and stress-buffering perspectives. Spiritual well-being reflects a person’s sense of meaning, inner coherence, and capacity to engage with existential uncertainty [12,13]. Perceived social support represents an interpersonal resource that may be associated with a greater sense of security and more adaptive appraisal of stressful experiences [16]. Previous research among pregnant women has shown that spiritual well-being and perceived social support are positively associated and that both are related to more favorable psychological outcomes [14,17]. On this basis, spiritual well-being was specified as the focal psychosocial resource, perceived social support as an intervening interpersonal variable, and death anxiety as the psychological outcome in the proposed analytical model. This ordering was specified a priori to examine one theoretically plausible pattern of associations rather than to establish a temporal or causal sequence. Spiritual well-being and perceived social support may be reciprocally related; for example, spiritual well-being may influence how available support is perceived, while supportive relationships may also strengthen spiritual well-being. Alternative directions are also possible, including the possibility that death anxiety influences perceptions of social support or spiritual well-being. Because all variables were measured concurrently, the present study cannot distinguish among these alternative temporal directions. The proposed model should therefore be interpreted as an exploratory cross-sectional indirect association model.

Therefore, this study aimed to examine the cross-sectional associations among spiritual well-being, perceived social support, and death anxiety in a sample of primigravid women. It also explored whether the observed association between spiritual well-being and death anxiety included a statistical indirect association through perceived social support. We hypothesized that (H1) higher spiritual well-being would be associated with lower death anxiety; (H2) higher spiritual well-being would be associated with greater perceived social support; (H3) greater perceived social support would be associated with lower death anxiety; and (H4) there would be a significant cross-sectional indirect association between spiritual well-being and death anxiety through perceived social support.

## 2. Materials and Methods

### 2.1. Study Design and Setting

This cross-sectional analytical study was conducted to examine the relationships between spiritual well-being, perceived social support, and death anxiety among primigravid women. Data were collected between February and May 2026 at the antenatal outpatient clinic of a training and research hospital providing specialized women’s and children’s health services in a relatively rural province in northern Türkiye. Approximately 2254 pregnant women attended the hospital’s antenatal outpatient clinic in 2025.

### 2.2. Participants and Sample Size

Participants were recruited using non-probability consecutive sampling from primigravid women attending the antenatal outpatient clinic between February and May 2026. During the recruitment period, 421 women were assessed for eligibility. Sixteen women did not meet the eligibility criteria. Of the 405 eligible women invited to participate, 15 declined and 390 agreed to participate, resulting in a response rate of 96.3%. Because the questionnaires were completed face-to-face and checked by the researcher immediately upon submission, no participants were excluded because of incomplete data. Recruitment ended at the conclusion of the predefined data-collection period in May 2026; therefore, the final sample of 390 represented the eligible women who agreed to participate during this period rather than a predetermined target for the indirect association analysis.

The minimum sample size was calculated using G*Power 3.1.9.7 [18] for the planned multiple linear regression analysis. The calculation was based on a medium effect size (f^2^ = 0.15), a significance level of α = 0.05, 90% statistical power, and six predictors, resulting in a minimum required sample of 123 participants. This calculation was specific to the multiple regression analysis and did not constitute an a priori power calculation for the indirect-effect analysis. No separate a priori sample-size or power calculation was performed for the indirect effect. The cross-sectional indirect association analysis was exploratory, and the statistical uncertainty of the indirect association was evaluated using 5000 bootstrap samples and 95% confidence intervals [19,20,21].

Women were eligible if they were aged ≥ 18 years, primigravid, had a singleton viable pregnancy, were at or beyond 14 weeks of gestation, were able to read and understand Turkish, and voluntarily agreed to participate. Women with a high-risk pregnancy or a physician-diagnosed psychiatric disorder were excluded. A high-risk pregnancy was defined as a pregnancy documented by a physician in the hospital records as high risk—such as a pregnancy complicated by a hypertensive disorder, pregestational or gestational diabetes, placenta previa, or threatened preterm birth—or requiring follow-up in the perinatology/high-risk pregnancy unit. Women who reported having a physician-diagnosed psychiatric disorder or had such a diagnosis documented in their medical records were also excluded.

### 2.3. Data Collection Procedure

Data were collected face-to-face at the antenatal outpatient clinic. Eligible women were informed about the purpose and procedures of the study before participation. After written informed consent was obtained, participants completed the data collection forms. Completion of the questionnaires took approximately 15–20 min. Data were collected using a Participant Information Form, the Multidimensional Scale of Perceived Social Support, the Three-Factor Spiritual Well-Being Scale, and the Death Anxiety Scale.

### 2.4. Participant Information Form

The Participant Information Form was developed by the researchers based on previous studies examining sociodemographic, obstetric, psychosocial, and spiritual characteristics of pregnant women [2,5,17]. It included questions on age, marital status, educational level, employment status, income level, family structure, place of residence, gestational week, whether the pregnancy was planned, and regular attendance at antenatal follow-up. The form also included questions addressing psychosocial and spiritual characteristics, including self-reported anxiety during pregnancy, perceived religiosity, the importance attributed to spirituality, the perceived effect of spirituality on pregnancy, and spiritual practices. The presence of a medical problem was determined by asking participants whether they had a current physician-diagnosed health condition and was coded dichotomously as “no” or “yes.” For this study, a medical problem referred to a self-reported physician-diagnosed health condition that did not meet the study definition of a high-risk pregnancy and did not require follow-up in the perinatology/high-risk pregnancy unit. Anxiety during pregnancy was assessed using the single self-reported item, “Overall, how anxious do you feel during your pregnancy?” The response options were “very little,” “little,” “moderate,” “high,” and “very high,” coded from 1 to 5, respectively. This researcher-developed item was used to obtain a brief subjective assessment of perceived anxiety during pregnancy. It was not a validated anxiety measure and was not intended to provide a clinical assessment or diagnosis of anxiety.

### 2.5. Multidimensional Scale of Perceived Social Support

Perceived social support was assessed using the Multidimensional Scale of Perceived Social Support (MSPSS), originally developed by Zimet et al. [22]. The validity and reliability of the Turkish version were established by Eker and Arkar [23]. The scale consists of 12 items covering three sources of perceived support: family, friends, and a significant other. Items are rated on a seven-point Likert scale, with total scores ranging from 12 to 84; higher scores indicate greater perceived social support. The Cronbach’s alpha coefficient for the Turkish version was reported as 0.89 [23]. In the present study, Cronbach’s alpha for the total scale was 0.88. All participants were married and had a spouse at the time of data collection. To standardize the referent across participants, women were instructed to consider their spouse when responding to the items of the “significant other” subscale. No participant was without a spouse; therefore, no alternative instructions were required. The wording, response options, and scoring of the original MSPSS items were not modified; only the referent for the “significant other” subscale was specified as the spouse. Nevertheless, the findings for this subscale should be interpreted specifically as perceived spousal support rather than support from a broadly defined significant other.

### 2.6. Three-Factor Spiritual Well-Being Scale

Spiritual well-being was assessed using the Three-Factor Spiritual Well-Being Scale developed by Ekşi and Kardaş [24]. The scale consists of 29 items across three dimensions: transcendence, harmony with nature, and anomie. Items are rated on a five-point Likert scale. Items in the anomie subscale are reverse-scored. Subscale and total scores are calculated as mean scores, with higher total mean scores indicating greater spiritual well-being. In the original development study, Cronbach’s alpha coefficients were 0.953 for transcendence, 0.864 for harmony with nature, 0.853 for anomie, and 0.886 for the total scale [24]. In the present study, Cronbach’s alpha was 0.93 for the total scale and 0.92, 0.86, and 0.85 for transcendence, harmony with nature, and anomie, respectively.

### 2.7. Death Anxiety Scale

Death anxiety was assessed using the Death Anxiety Scale (DAS) developed and validated in Turkish by Sarıkaya and Baloğlu [11]. The scale contains 20 items across three dimensions: uncertainty of death, thinking about and witnessing death, and suffering. Items are scored from 0 to 4, producing a total score ranging from 0 to 80, with higher scores indicating greater death anxiety. Scores of 0–29, 30–59, and 60–80 are interpreted as low, moderate, and high levels of death anxiety, respectively [11]. In the original study, Cronbach’s alpha was 0.95 for the total scale and 0.94, 0.92, and 0.76 for the uncertainty of death, thinking about and witnessing death, and suffering subscales, respectively [11]. In the present study, Cronbach’s alpha was 0.93 for the total scale and 0.90, 0.88, and 0.84 for the three respective subscales.

### 2.8. Statistical Analysis

Data were analyzed using IBM SPSS Statistics, version 26.0 (IBM Corp., Armonk, NY, USA). Descriptive statistics are presented as frequencies and percentages for categorical variables and means and standard deviations for continuous variables. The distribution of continuous variables was evaluated using skewness and kurtosis values.

Before conducting the hierarchical multiple linear regression analysis, the assumptions of linearity, normality, homoscedasticity, independence of errors, and multicollinearity were checked. Multicollinearity was assessed using tolerance and variance inflation factor (VIF) values, and the Durbin–Watson statistic was used to examine the independence of errors. Standardized residuals and residual plots were also examined for normality, linearity, and homoscedasticity. The results indicated no substantial violations of these assumptions.

Pearson’s correlation analysis was used to examine the relationships among spiritual well-being, perceived social support, and death anxiety. Covariate selection for the revised hierarchical regression model was guided by a biopsychosocial framework and previous evidence indicating that sociodemographic, obstetric, medical, and psychological characteristics may be associated with perceived social support and anxiety-related outcomes during pregnancy [3,14,16,17]. Age, gestational week, the presence of a medical problem, planned pregnancy, educational level, employment status, and income level were entered as background covariates in Model 1. Self-reported anxiety during pregnancy was added in Model 2 as a competing psychological correlate. Spiritual well-being and perceived social support, the focal variables of the study, were entered in Model 3 to examine their additional contribution after adjustment for the specified covariates. Educational level, income level, and self-reported anxiety during pregnancy were represented using indicator variables. University education or higher, low income, and “very little” anxiety were used as the respective reference categories. Unemployment, no medical problem, and an unplanned pregnancy were used as the reference categories for the binary variables. There were no missing data among the 390 participants included in the analysis; therefore, no imputation was performed.

An exploratory cross-sectional indirect association analysis was conducted using PROCESS macro for SPSS, version 4.2 [19]. Spiritual well-being was specified as X, perceived social support as the intervening variable (M), and death anxiety as Y. The model included age, gestational week, the presence of a medical problem, planned pregnancy, educational level, employment status, income level, and self-reported anxiety during pregnancy as covariates. Multicategorical covariates were entered using the same indicator coding and reference categories as in the hierarchical regression analysis. This specification was used to estimate the adjusted statistical indirect association and did not imply temporal ordering or causality. The indirect association was estimated using 5000 bootstrap samples with 95% confidence intervals (CIs) and was considered statistically significant when the bootstrap CI did not include zero [19,21]. Statistical significance was set at *p* < 0.05.

### 2.9. Ethical Considerations

The study was conducted in accordance with the principles of the Declaration of Helsinki. Ethical approval was granted by the Giresun University Social Sciences, Science and Engineering Research Ethics Committee (Decision No. E-50288587-050.01.04-145472; January 2026), and institutional permission was obtained from the hospital where the study was conducted. Written informed consent was obtained from all participants before data collection, and their privacy and confidentiality were protected throughout the study.

## 3. Results

### 3.1. Participant Characteristics

A total of 390 primigravid women were included in the study. The mean age of the participants was 27.09 ± 4.21 years, and the mean gestational week was 24.09 ± 7.26. Most participants had completed at least high school education; 59.0% were unemployed, and 61.3% reported a moderate income level. Most lived in nuclear families (71.8%), and 52.8% lived in the city center. The majority reported a planned pregnancy (76.9%) and regular antenatal follow-up (86.7%). Most participants reported no current medical problem (81.8%), and moderate self-reported anxiety during pregnancy was the most frequently reported category (28.5%) (Table 1).

### 3.2. Descriptive Statistics of the Study Variables

Descriptive statistics for the study measures are presented in Table 2. The mean spiritual well-being score was 3.17 ± 0.81. Mean scores for harmony with nature, transcendence, and anomie were 3.21 ± 0.66, 3.09 ± 0.95, and 2.72 ± 0.85, respectively. The mean perceived social support score was 50.69 ± 13.76, with mean subscale scores of 17.30 ± 5.78 for spousal support, 17.23 ± 4.92 for family support, and 16.15 ± 4.41 for friend support. The mean death anxiety score was 37.97 ± 16.68. The corresponding mean subscale scores were 19.23 ± 9.20 for uncertainty of death, 13.17 ± 6.04 for thinking about and witnessing death, and 5.57 ± 2.65 for suffering (Table 2).

### 3.3. Correlations Among Spiritual Well-Being, Perceived Social Support, and Death Anxiety

The correlations among the main study variables are presented in Table 3. Spiritual well-being was positively correlated with perceived social support (r = 0.495, *p* < 0.001) and negatively correlated with death anxiety (r = −0.554, *p* < 0.001). Perceived social support was also negatively correlated with death anxiety (r = −0.522, *p* < 0.001).

### 3.4. Hierarchical Regression Analysis of Death Anxiety

The results of the hierarchical multiple linear regression analysis are presented in Table 4. Model 1 included age, gestational week, the presence of a medical problem, planned pregnancy, educational level, employment status, and income level. The model accounted for 4.4% of the variance in death anxiety (R^2^ = 0.044; adjusted R^2^ = 0.018) and was not statistically significant overall, F(10, 379) = 1.73, *p* = 0.073. Among the variables entered, only the presence of a medical problem was positively associated with death anxiety (B = 6.53, SE = 2.18, 95% CI [2.25, 10.80], β = 0.151, *p* = 0.003). Age, gestational week, planned pregnancy, educational level, employment status, and income level were not statistically significant.

In Model 2, self-reported anxiety during pregnancy was added using indicator variables, with “very little” anxiety as the reference category. This addition significantly increased the variance accounted for by 64.3%, ΔR^2^ = 0.643, ΔF(4, 375) = 192.50, *p* < 0.001. Compared with women reporting very little anxiety, death anxiety scores were significantly higher among women reporting little anxiety (B = 12.62, SE = 1.65, 95% CI [9.38, 15.86], β = 0.306, *p* < 0.001), moderate anxiety (B = 22.74, SE = 1.56, 95% CI [19.68, 25.81], β = 0.616, *p* < 0.001), high anxiety (B = 32.91, SE = 1.61, 95% CI [29.73, 36.08], β = 0.835, *p* < 0.001), or very high anxiety (B = 45.37, SE = 1.88, 95% CI [41.67, 49.07], β = 0.903, *p* < 0.001). None of the other covariates was statistically significant in Model 2. This model accounted for 68.7% of the variance in death anxiety (R^2^ = 0.687; adjusted R^2^ = 0.675), F(14, 375) = 58.73, *p* < 0.001.

In Model 3, spiritual well-being and perceived social support were added as the focal variables. Their addition produced a further significant increase of 4.0% in the variance accounted for by the model, ΔR^2^ = 0.040, ΔF(2, 373) = 27.55, *p* < 0.001. Spiritual well-being (B = −3.12, SE = 0.70, 95% CI [−4.50, −1.74], β = −0.152, *p* < 0.001) and perceived social support (B = −0.18, SE = 0.04, 95% CI [−0.25, −0.10], β = −0.145, *p* < 0.001) were both negatively associated with death anxiety after adjustment for all prespecified covariates. The presence of a medical problem also remained positively associated with death anxiety (B = 2.89, SE = 1.20, 95% CI [0.53, 5.25], β = 0.067, *p* = 0.017). The indicator variables representing self-reported anxiety during pregnancy remained statistically significant, whereas age, gestational week, planned pregnancy, educational level, employment status, and income level were not statistically significant in the final model.

The final model accounted for 72.7% of the variance in death anxiety (R^2^ = 0.727; adjusted R^2^ = 0.715), F(16, 373) = 62.11, *p* < 0.001. No evidence of problematic multicollinearity was observed, with tolerance values ranging from 0.41 to 0.97 and VIF values ranging from 1.03 to 2.46. The Durbin–Watson statistic was 1.97, indicating no apparent concern regarding the independence of residuals.

### 3.5. Exploratory Cross-Sectional Indirect Association Analysis

**Figure 1:**
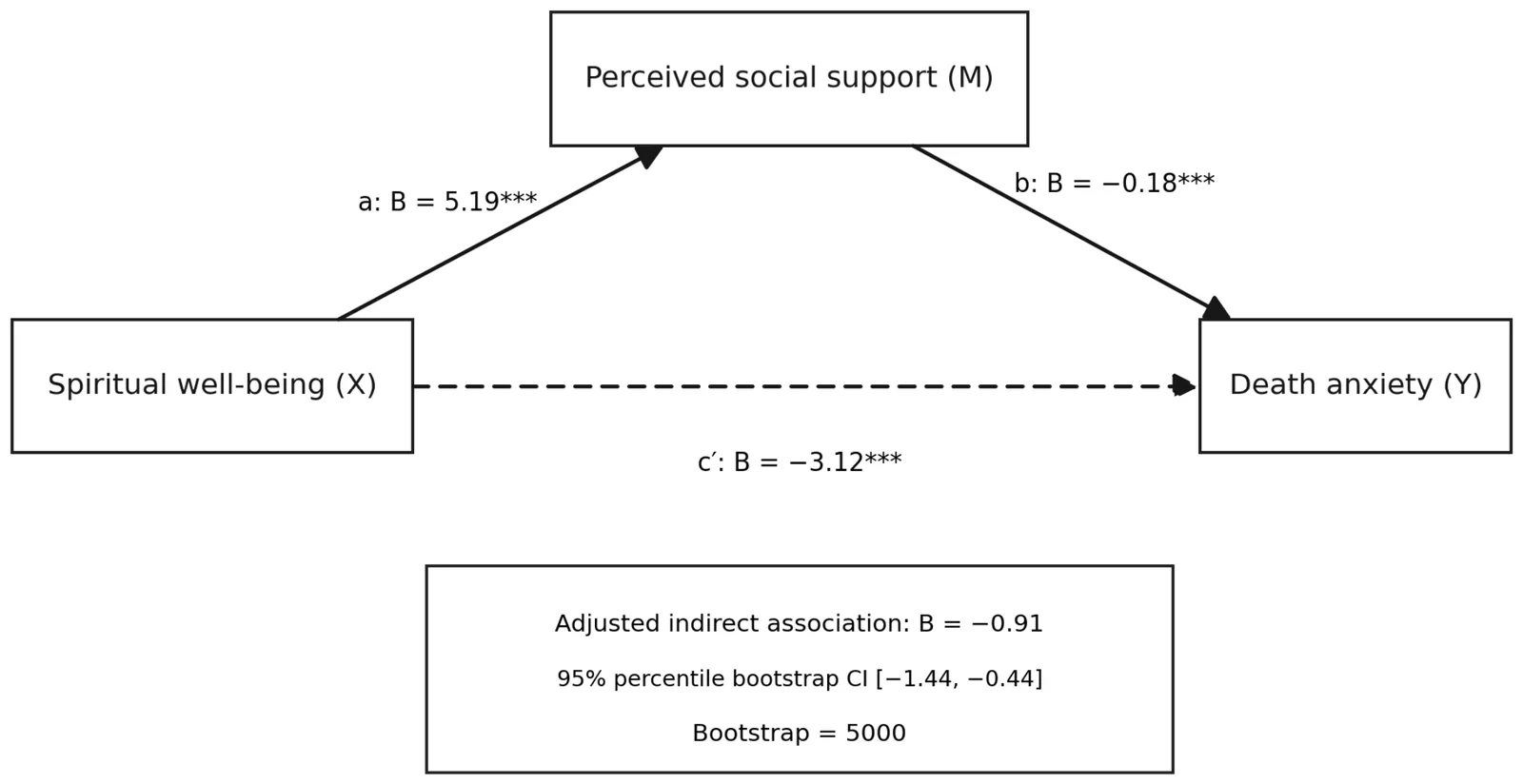
Adjusted exploratory cross-sectional indirect association model of spiritual well-being, perceived social support, and death anxiety. Note. Values represent unstandardized regression coefficients adjusted for age, gestational week, the presence of a medical problem, planned pregnancy, educational level, employment status, income level, and self-reported anxiety during pregnancy. Multicategorical covariates were entered using indicator variables. The adjusted indirect association was estimated using 5000 bootstrap samples and a percentile bootstrap 95% confidence interval. The arrows represent the statistical specification of the cross-sectional model and do not demonstrate temporal ordering or causal direction. *** *p* < 0.001.

The adjusted exploratory cross-sectional indirect association analysis is presented in Table 5 and Figure 1. The model was adjusted for age, gestational week, the presence of a medical problem, planned pregnancy, educational level, employment status, income level, and self-reported anxiety during pregnancy. Spiritual well-being was positively associated with perceived social support (path a: B = 5.19, SE = 0.86, 95% CI [3.49, 6.89], *p* < 0.001). Perceived social support was negatively associated with death anxiety after adjustment for spiritual well-being and the specified covariates (path b: B = −0.18, SE = 0.04, 95% CI [−0.25, −0.10], *p* < 0.001).

The total adjusted association between spiritual well-being and death anxiety was statistically significant (path c: B = −4.03, SE = 0.69, 95% CI [−5.38, −2.68], *p* < 0.001). After perceived social support was included in the model, the adjusted direct association remained statistically significant (path c′: B = −3.12, SE = 0.70, 95% CI [−4.50, −1.74], *p* < 0.001).

A statistically significant adjusted indirect effect was observed in the specified cross-sectional model (B = −0.91, 95% percentile bootstrap CI [−1.44, −0.44]). The confidence interval was estimated using 5000 bootstrap samples and did not include zero. This finding represents a statistical indirect association within the cross-sectional data and does not establish mediation, temporal ordering, or a causal pathway. Similarly, the arrows shown in Figure 1 represent the statistical specification of the tested model and should not be interpreted as evidence of temporal or causal direction.

## 4. Discussion

This study examined the cross-sectional associations among spiritual well-being, perceived social support, and death anxiety in primigravid women. Higher spiritual well-being and greater perceived social support were associated with lower death anxiety. These associations remained statistically significant after adjustment for age, gestational week, the presence of a medical problem, planned pregnancy, educational level, employment status, income level, and self-reported anxiety during pregnancy. Spiritual well-being was also positively associated with perceived social support. In addition, a statistically significant adjusted indirect association through perceived social support was observed in the specified cross-sectional model. Overall, the findings were consistent with the hypothesized associations; however, they do not establish temporal ordering or causality and cannot demonstrate that death anxiety was attributable to or heightened by a first pregnancy.

The participants had a moderate level of death anxiety, with uncertainty of death having the highest mean score among the subscales. Although pregnancy may involve uncertainty related to physical changes, fetal health, childbirth, and possible maternal or fetal complications, the Death Anxiety Scale used in this study assesses general mortality-related thoughts and emotions rather than pregnancy-specific fears or mortality concerns [11]. Therefore, the higher score for uncertainty of death should be interpreted as reflecting general uncertainty concerning mortality rather than as evidence of anxiety arising specifically from pregnancy. Previous research has linked death anxiety with anxiety and broader psychological distress [7]. In this context, our findings suggest that death anxiety may be a relevant aspect of psychological well-being among primigravid women. However, because no multigravid or non-pregnant comparison group was included, the findings cannot determine whether death anxiety is specifically attributable to or heightened by a first pregnancy.

As hypothesized, women with higher spiritual well-being reported lower death anxiety. This finding is broadly consistent with studies reporting associations between spiritual well-being and lower pregnancy-related anxiety and stress [14,25], between positive religious attitudes and lower anxiety and greater psychological well-being [26], and between religiosity or spiritual coping and maternal mental health [27,28]. However, these studies examined pregnancy-related distress and other psychological outcomes rather than death anxiety. Spiritual well-being may be related to how women interpret difficult or uncertain experiences and to their reported sense of meaning and inner stability. Nevertheless, the present cross-sectional findings cannot establish that spiritual well-being reduces death anxiety or that this association is specific to first pregnancy. However, the cross-sectional nature of our data does not allow us to conclude that spiritual well-being leads to lower death anxiety.

Perceived social support was also associated with lower death anxiety. Although evidence specifically linking social support to death anxiety during pregnancy is limited, a systematic review showed that stronger social support during pregnancy was associated with greater subjective well-being and a lower risk of psychological distress, including depression [29]. Having someone with whom to share concerns and receiving emotional or practical support may be relevant to psychological well-being during pregnancy. In the present sample, perceived social support was also associated with lower general death anxiety.

Spiritual well-being was positively associated with perceived social support, which was also in line with our hypothesis. Similar findings have been reported in pregnant women, including women with high-risk pregnancies [14,17]. One possible explanation is that women with greater spiritual well-being may view their relationships and available support more positively. It is also possible that supportive relationships strengthen a woman’s sense of meaning and spiritual well-being. The present study cannot establish which direction is more likely, and the relationship may in fact be reciprocal.

In the hierarchical regression analysis, the sociodemographic and obstetric covariates entered in Model 1 accounted for 4.4% of the variance in death anxiety, although the model was not statistically significant overall. The presence of a medical problem was the only significant variable at this stage. When self-reported anxiety during pregnancy was added as indicator variables in Model 2, the model accounted for 68.7% of the variance. Compared with women reporting very little anxiety, those reporting progressively higher anxiety levels also had progressively higher death anxiety scores. This finding is consistent with the established relationship between death anxiety and broader anxiety-related distress [7]. Nevertheless, the substantial contribution of this variable should be interpreted cautiously because anxiety during pregnancy was assessed using a single researcher-developed self-report item rather than a validated anxiety instrument. It should therefore not be regarded as equivalent to adjustment using a validated measure of anxiety.

The addition of spiritual well-being and perceived social support in Model 3 accounted for a further 4.0% of the variance in death anxiety, and the final model accounted for 72.7% of the variance. Both spiritual well-being and perceived social support remained negatively associated with death anxiety after adjustment for the prespecified sociodemographic, obstetric, and anxiety-related covariates. The presence of a medical problem was also positively associated with death anxiety in the final model, whereas age, gestational week, planned pregnancy, educational level, employment status, and income level were not statistically significant. The association between self-reported medical problems and death anxiety may reflect a greater perception of vulnerability among women experiencing health concerns during pregnancy. More broadly, physical health conditions and comorbidities have been identified as factors relevant to maternal psychological well-being during pregnancy [30]. However, because the type and severity of the reported medical problems were not clinically assessed in the present study, this finding should be interpreted cautiously.

A statistically significant adjusted indirect association through perceived social support was observed in the specified cross-sectional model. Spiritual well-being was positively associated with perceived social support, which was in turn negatively associated with death anxiety after adjustment for the prespecified covariates. The adjusted direct association between spiritual well-being and death anxiety also remained statistically significant when perceived social support was included in the model. This pattern was consistent with the hypothesized indirect association. Grzesik-Gąsior et al. [31] similarly reported an indirect role of social support in the relationship between pregnancy anxiety and emotional processes. The present findings extend this literature by identifying a statistical indirect association among spiritual well-being, perceived social support, and death anxiety.

However, the ordering specified in PROCESS represents a theoretically proposed statistical model rather than an established temporal sequence. Because all variables were measured concurrently, the analysis cannot determine whether spiritual well-being preceded perceived social support or whether perceived social support preceded death anxiety. Alternative models—for example, one in which lower death anxiety is associated with higher perceived social support, or one in which perceived social support precedes spiritual well-being—may be statistically equivalent or indistinguishable in cross-sectional data. Reciprocal relationships are also possible. Therefore, the observed indirect association should not be interpreted as evidence of mediation or a causal pathway.

From a clinical perspective, the findings provide exploratory evidence that spiritual well-being and perceived social support may be relevant when considering the psychosocial experiences of primigravid women. Midwives and nurses may consider asking women about available sources of support and, when appropriate, whether spiritual concerns are personally important to them. However, the present findings do not support routine clinical recommendations, demonstrate that spiritual or social-support interventions reduce death anxiety, or establish that the observed death anxiety arose from pregnancy. The potential clinical relevance of these factors should therefore be examined in longitudinal and intervention studies using validated measures of pregnancy-specific anxiety, fear of childbirth, and mortality-related concerns.

### Strengths and Limitations

A strength of this study is its relatively large sample of primigravid women and its simultaneous examination of spiritual, social, and psychological factors. The hierarchical regression analysis incorporated prespecified sociodemographic, obstetric, and anxiety-related covariates, while the bootstrap-based exploratory analysis allowed the statistical uncertainty surrounding the adjusted indirect association to be evaluated. The study also provides information about general death anxiety in a clearly defined antenatal population that has received limited attention in previous research.

This study has several important limitations. First, its cross-sectional design does not permit conclusions regarding causality or the temporal ordering of the observed relationships. Although spiritual well-being was specified as X, perceived social support as the intervening variable (M), and death anxiety as Y, this ordering reflects the proposed conceptual framework rather than an empirically demonstrated temporal sequence. Alternative orderings of these variables may produce statistically equivalent or indistinguishable models in cross-sectional data. For example, lower death anxiety may be associated with higher perceived social support, or perceived social support may precede spiritual well-being. Reciprocal relationships are also possible. Accordingly, the statistically significant indirect association should not be interpreted as evidence of mediation or a causal pathway.

Second, participants were recruited using non-probability consecutive sampling from a single hospital located in a relatively rural province of northern Türkiye. This recruitment strategy may have introduced selection bias because women attending this hospital and agreeing to participate may differ systematically from women receiving care in other settings or from those who do not attend antenatal services. Moreover, spiritual well-being and perceived social support are likely to be influenced by cultural, familial, religious, regional, socioeconomic, and healthcare contexts. The external validity of the findings is therefore substantially constrained, and the results should not be generalized to primigravid women in other settings without caution.

Third, the study did not include multigravid or non-pregnant comparison groups. It therefore cannot determine whether death anxiety was heightened by or attributable to a first pregnancy. Furthermore, the Death Anxiety Scale assesses general death anxiety rather than pregnancy-specific mortality concerns, pregnancy-related anxiety, fear of childbirth, or concerns about maternal and fetal complications. The findings should consequently be interpreted as associations observed in a sample of primigravid women rather than as evidence of death anxiety caused specifically by first pregnancy.

Fourth, although the analyses were adjusted for age, gestational week, the presence of a medical problem, planned pregnancy, educational level, employment status, income level, and self-reported anxiety during pregnancy, residual and unmeasured confounding remains possible. Factors such as previous reproductive loss, exposure to death or serious illness, stressful life events, relationship quality, and other mental health characteristics were not measured and may have influenced the observed associations.

Fifth, anxiety during pregnancy was assessed using a single researcher-developed self-report item rather than a validated anxiety instrument. Although indicator coding avoided assuming equal distances between the response categories, this item provides only a subjective assessment and should not be interpreted as a clinical or psychometrically validated measure of anxiety. The substantial increase in the variance accounted for after this variable was added to the regression model should therefore be interpreted cautiously.

Sixth, spiritual well-being, perceived social support, death anxiety, and self-reported anxiety during pregnancy were assessed concurrently using the same self-report method. This creates a risk of common-method bias arising from shared response tendencies, social desirability, and the common measurement context. Consequently, the magnitudes of the observed correlations and regression associations may have been inflated or otherwise influenced by method-related variance. There may also be conceptual overlap between psychological distress and some aspects of spiritual well-being, particularly those concerning meaning, harmony, and anomie. Although the instruments were designed to assess distinct constructs, this potential overlap may have contributed to the magnitude of the observed associations.

Finally, women with physician-diagnosed psychiatric disorders or high-risk pregnancies were excluded. The findings therefore cannot be generalized to these groups, who may have different levels of death anxiety, spiritual well-being, perceived social support, or broader psychological distress.

Longitudinal research assessing spiritual well-being, perceived social support, and death anxiety at multiple points during pregnancy and the postpartum period is needed to clarify temporal ordering and potentially reciprocal relationships. Future studies should include multigravid and non-pregnant comparison groups and use validated measures that distinguish general death anxiety from pregnancy-related anxiety, fear of childbirth, and pregnancy-specific mortality concerns. Multicenter studies using probability-based sampling across different geographical, cultural, and healthcare settings would improve external validity. The use of temporally separated assessments and multiple measurement methods or data sources may also help reduce common-method bias. Finally, intervention studies are required before concluding that strengthening social support or addressing women’s individual spiritual needs can reduce death anxiety or related psychological outcomes.

## 5. Conclusions

Higher spiritual well-being and perceived social support were associated with lower death anxiety among primigravid women. A statistically significant adjusted indirect association through perceived social support was observed in the specified cross-sectional model. However, the cross-sectional design does not establish mediation, temporal ordering, or causality, and alternative or reciprocal directions among the variables remain possible.

These findings provide exploratory evidence that spiritual and social resources may be relevant to the psychosocial experiences of primigravid women. Because no multigravid or non-pregnant comparison group was included and the Death Anxiety Scale measures general death anxiety, the findings cannot demonstrate that death anxiety was caused or heightened by first pregnancy. Longitudinal, multicenter, and intervention studies are needed to clarify the temporal relationships and clinical relevance of these findings.

## Figures and Tables

**Table 1:** Sociodemographic, Obstetric, and Psychosocial Characteristics of Participants (*n* = 390).

Characteristic	Mean ± SD	Min–Max
Age (years)	27.09 ± 4.21	18–40
Gestational week	24.09 ± 7.26	14–40
Educational level	***n*** **(%)**	
Primary school	41 (10.5)	
Middle school	84 (21.5)	
High school	142 (36.4)	
University or higher	123 (31.5)	
Employment status		
Employed	160 (41.0)	
Unemployed	230 (59.0)	
Income level		
Low	90 (23.1)	
Moderate	239 (61.3)	
High	61 (15.6)	
Family structure		
Nuclear family	280 (71.8)	
Extended family	110 (28.2)	
Place of residence		
City center	206 (52.8)	
District	132 (33.8)	
Village/rural area	52 (13.3)	
Planned pregnancy		
Yes	300 (76.9)	
No	90 (23.1)	
Regular antenatal follow-up		
Yes	338 (86.7)	
No	52 (13.3)	
Presence of a medical problem		
Yes	71 (18.2)	
No	319 (81.8)	
Self-reported anxiety level during pregnancy		
Very little	59 (15.1)	
Little	80 (20.5)	
Moderate	111 (28.5)	
High	91 (23.3)	
Very high	49 (12.6)	
Marital status		
Married	390 (100.0)	

**Table 2:** Descriptive Statistics for the Study Measures (*n* = 390).

Scale/Subscale	Minimum	Maximum	Mean ± SD
Three-Factor Spiritual Well-Being Scale			
Transcendence	1.00	5.00	3.09 ± 0.95
Harmony with nature	1.00	5.00	3.21 ± 0.66
Anomie	1.00	5.00	2.72 ± 0.85
Total	1.14	5.00	3.17 ± 0.81
Multidimensional Scale of Perceived Social Support (MSPSS)			
Spousal support (MSPSS significant other subscale)	4.00	28.00	17.30 ± 5.78
Family	6.00	28.00	17.23 ± 4.92
Friends	4.00	28.00	16.15 ± 4.41
Total	18.00	81.00	50.69 ± 13.76
Death Anxiety Scale (DAS)			
Uncertainty of death	0.00	40.00	19.23 ± 9.20
Thinking about and witnessing death	0.00	28.00	13.17 ± 6.04
Suffering	0.00	12.00	5.57 ± 2.65
Total	2.00	79.00	37.97 ± 16.68

Note: Minimum and maximum values represent the observed scores in the study sample. SD, standard deviation. All participants were married and were instructed to consider their spouse when responding to the MSPSS significant other subscale.

**Table 3:** Correlations among the Study Measures (*n* = 390).

Measure	1	2	3
1. Three-Factor Spiritual Well-Being Scale	1		
2. Multidimensional Scale of Perceived Social Support (MSPSS)	0.495 ***	1	
3. Death Anxiety Scale (DAS)	−0.554 ***	−0.522 ***	1

Note: Values are Pearson’s correlation coefficients. *** *p* < 0.001.

**Table 4:** Hierarchical Multiple Linear Regression Analysis Predicting Death Anxiety (*n* = 390).

**Model 1**					
**Variable**	**B**	**SE**	**95% CI for B**	**β**	***p***
Age	0.00	0.20	[−0.39, 0.40]	0.000	0.998
Gestational week	0.12	0.13	[−0.13, 0.37]	0.047	0.356
Medical problem: Yes	6.53	2.18	[2.25, 10.80]	0.151	0.003
Planned pregnancy: Yes	−3.02	2.03	[−7.01, 0.96]	−0.076	0.136
Education: High school	−0.38	2.05	[−4.40, 3.65]	−0.011	0.855
Education: Middle school	2.61	2.34	[−1.99, 7.22]	0.064	0.265
Education: Primary school	2.69	3.00	[−3.22, 8.60]	0.050	0.371
Employment: Employed	1.19	1.72	[−2.19, 4.57]	0.035	0.490
Income: Moderate	−2.24	2.06	[−6.30, 1.82]	−0.066	0.278
Income: High	−4.28	2.77	[−9.74, 1.17]	−0.093	0.123
R^2^	0.044				
Adjusted R^2^	0.018				
Model F	1.73 (*p* = 0.073)				
**Model 2**					
**Variable**	**B**	**SE**	**95% CI for B**	**β**	***p***
Age	0.08	0.12	[−0.15, 0.31]	0.020	0.487
Gestational week	0.04	0.07	[−0.10, 0.19]	0.018	0.552
Medical problem: Yes	1.55	1.27	[−0.95, 4.04]	0.036	0.224
Planned pregnancy: Yes	0.49	1.18	[−1.83, 2.80]	0.012	0.680
Education: High school	1.33	1.18	[−0.99, 3.66]	0.038	0.260
Education: Middle school	1.51	1.35	[−1.15, 4.17]	0.037	0.265
Education: Primary school	1.63	1.74	[−1.78, 5.04]	0.030	0.348
Employment: Employed	0.12	0.99	[−1.83, 2.07]	0.004	0.904
Income: Moderate	−0.71	1.20	[−3.06, 1.64]	−0.021	0.553
Income: High	−0.52	1.61	[−3.68, 2.65]	−0.011	0.748
Anxiety: Little	12.62	1.65	[9.38, 15.86]	0.306	<0.001
Anxiety: Moderate	22.74	1.56	[19.68, 25.81]	0.616	<0.001
Anxiety: High	32.91	1.61	[29.73, 36.08]	0.835	<0.001
Anxiety: Very high	45.37	1.88	[41.67, 49.07]	0.903	<0.001
R^2^	0.687				
Adjusted R^2^	0.675				
ΔR^2^	0.643				
ΔF	192.50 (*p* < 0.001)				
Model F	58.73 (*p* < 0.001)				
**Model 3**					
**Variable**	**B**	**SE**	**95% CI for B**	**β**	***p***
Age	0.06	0.11	[−0.16, 0.27]	0.014	0.606
Gestational week	0.04	0.07	[−0.10, 0.17]	0.016	0.574
Medical problem: Yes	2.89	1.20	[0.53, 5.25]	0.067	0.017
Planned pregnancy: Yes	−0.43	1.11	[−2.61, 1.75]	−0.011	0.698
Education: High school	0.50	1.11	[−1.69, 2.68]	0.014	0.656
Education: Middle school	0.00	1.28	[−2.52, 2.53]	0.000	0.998
Education: Primary school	0.38	1.63	[−2.83, 3.59]	0.007	0.816
Employment: Employed	−0.54	0.93	[−2.37, 1.29]	−0.016	0.562
Income: Moderate	0.36	1.13	[−1.86, 2.58]	0.011	0.750
Income: High	1.12	1.52	[−1.87, 4.12]	0.024	0.461
Anxiety: Little	12.04	1.54	[9.01, 15.07]	0.292	<0.001
Anxiety: Moderate	19.80	1.51	[16.83, 22.77]	0.536	<0.001
Anxiety: High	27.62	1.67	[24.33, 30.91]	0.701	<0.001
Anxiety: Very high	37.96	2.02	[33.98, 41.94]	0.755	<0.001
Spiritual well-being	−3.12	0.70	[−4.50,−1.74]	−0.152	<0.001
Perceived social support	−0.18	0.04	[−0.25,−0.10]	−0.145	<0.001
R^2^	0.727				
Adjusted R^2^	0.715				
ΔR^2^	0.040				
ΔF	27.55 (*p* < 0.001)				
Model F	62.11 (*p* < 0.001)				
**VIF values for Model 3**					
**Variable**	**VIF**				
Age	1.03				
Gestational week	1.05				
Medical problem: Yes	1.06				
Planned pregnancy: Yes	1.08				
Education: High school	1.41				
Education: Middle school	1.37				
Education: Primary school	1.24				
Employment: Employed	1.03				
Income: Moderate	1.49				
Income: High	1.51				
Anxiety: Little	1.91				
Anxiety: Moderate	2.29				
Anxiety: High	2.46				
Anxiety: Very high	2.21				
Spiritual well-being	1.61				
Perceived social support	1.50				

Note: B = unstandardized regression coefficient; SE = standard error; CI = confidence interval; β = standardized regression coefficient; VIF = variance inflation factor. Reference categories were university education or higher, unemployed, low income, no medical problem, unplanned pregnancy, and very little anxiety. Model 1 included sociodemographic and obstetric covariates. Model 2 additionally included self-reported anxiety during pregnancy as indicator variables. Model 3 additionally included spiritual well-being and perceived social support. Values smaller than 0.001 are reported as *p* < 0.001.

**Table 5:** Adjusted Exploratory Cross-Sectional Indirect Association Analysis of Spiritual Well-Being, Perceived Social Support, and Death Anxiety (*n* = 390).

Path/Association	B	SE	95% CI	*p*
Spiritual well-being–perceived social support association (*a*)	5.19	0.86	[3.49, 6.89]	<0.001
Perceived social support–death anxiety association (*b*)	−0.18	0.04	[−0.25, −0.10]	<0.001
Spiritual well-being–death anxiety total association (*c*)	−4.03	0.69	[−5.38, −2.68]	<0.001
Spiritual well-being–death anxiety direct association (*c*′)	−3.12	0.70	[−4.50, −1.74]	<0.001
Adjusted indirect association (*a* × *b*)	−0.91	—	[−1.44, −0.44] ^a^	—

Note: B = unstandardized regression coefficient; SE = standard error; CI = confidence interval. All estimates were adjusted for age, gestational week, the presence of a medical problem, planned pregnancy, educational level, employment status, income level, and self-reported anxiety during pregnancy. Multicategorical covariates were entered using indicator variables. ^a^ The confidence interval for the adjusted indirect association is a percentile bootstrap 95% confidence interval estimated using 5000 bootstrap samples. The adjusted indirect association was considered statistically significant because the bootstrap confidence interval did not include zero. The confidence intervals for paths *a*, *b*, *c*, and *c*′ are ordinary model-based 95% confidence intervals. Because all variables were measured concurrently, the specified indirect association does not establish mediation, temporal ordering, or causality.

## Data Availability

Due to ethical restrictions, the data are not publicly available but may be obtained from the corresponding author upon reasonable request.

## References

[1] Biaggi A., Conroy S., Pawlby S., Pariante C.M. (2016). Identifying the women at risk of antenatal anxiety and depression: A systematic review. J. Affect. Disord..

[2] Bishaw K.A., Andalem A., Amha H., Wondie T. (2022). Generalized anxiety disorder and its associated factors among pregnant women during COVID-19 at public health facilities of East Gojjam Zone, 2020: A multi-center cross-sectional study. Front. Glob. Womens Health.

[3] Jiang L., Zhu Z. (2022). Maternal mental health and social support from online communities during pregnancy. Health Soc. Care Community.

[4] Caffieri A., Gómez-Gómez I., Barquero-Jimenez C., De-Juan-Iglesias P., Margherita G., Motrico E. (2024). Global prevalence of perinatal depression and anxiety during the COVID-19 pandemic: An umbrella review and meta-analytic synthesis. Acta Obstet. Gynecol. Scand..

[5] Meng W., Shalayiding S., Wang X., Sailike B., Jiang T. (2025). Relationship between prenatal anxiety, depression, pregnancy stress and their social determinants. BMC Psychol..

[6] Menzies R.E., Menzies R.G. (2020). Death anxiety in the time of COVID-19: Theoretical explanations and clinical implications. Cogn. Behav. Ther..

[7] Menzies R.E., McMullen K., Riotto G.D., Iliescu S., Petrovic B., Remfrey M. (2024). From dread to disorder: A meta-analysis of the impact of death anxiety on mental illness symptoms. Clin. Psychol. Rev..

[8] Bayrampour H., Ali E., McNeil D.A., Benzies K., MacQueen G., Tough S. (2016). Pregnancy-related anxiety: A concept analysis. Int. J. Nurs. Stud..

[9] Ali M.H., Seif S.A., Kibusi S.M. (2022). The influence of fear during pregnancy, labour and delivery on birth outcome among post-delivery women: A case-control study in Zanzibar. East Afr. Health Res. J..

[10] Sejrsgaard M., Hvidtjørn D., Prinds C. (2025). The paradox of awareness of death in parenthood transition—A qualitative study. Death Stud..

[11] Sarıkaya Y., Baloğlu M. (2016). The development and psychometric properties of the Turkish Death Anxiety Scale (TDAS). Death Stud..

[12] Garssen B., Visser A., Pool G. (2021). Does spirituality or religion positively affect mental health? Meta-analysis of longitudinal studies. Int. J. Psychol. Relig..

[13] Koenig H.G. (2020). Maintaining health and well-being by putting faith into action during the COVID-19 pandemic. J. Relig. Health.

[14] Chehrazi M., Faramarzi M., Abdollahi S., Esfandiari M., Shafie Rizi S. (2021). Health promotion behaviours of pregnant women and spiritual well-being: Mediatory role of pregnancy stress, anxiety and coping ways. Nurs. Open.

[15] Keten Edis E., Bal S. (2024). Life satisfaction, psychological resilience, and spiritual well-being levels of pregnant women. Spirit. Psychol. Couns..

[16] Chen Z., Li Y., Chen J., Guo X. (2022). The mediating role of coping styles in the relationship between perceived social support and antenatal depression among pregnant women: A cross-sectional study. BMC Pregnancy Childbirth.

[17] Kiyak S. (2025). The relationship between fetal health anxiety, spiritual well-being, and perceived social support in high-risk pregnant women in Türkiye. J. Relig. Health.

[18] Faul F., Erdfelder E., Buchner A., Lang A.-G. (2009). Statistical power analyses using G*Power 3.1: Tests for correlation and regression analyses. Behav. Res. Methods.

[19] Hayes A.F. (2022). Introduction to Mediation, Moderation, and Conditional Process Analysis: A Regression-Based Approach.

[20] MacKinnon D.P. (2012). Introduction to Statistical Mediation Analysis.

[21] Preacher K.J., Hayes A.F. (2008). Asymptotic and resampling strategies for assessing and comparing indirect effects in multiple mediator models. Behav. Res. Methods.

[22] Zimet G.D., Dahlem N.W., Zimet S.G., Farley G.K. (1988). The Multidimensional Scale of Perceived Social Support. J. Pers. Assess..

[23] Eker D., Arkar H. (1995). Factorial structure, validity, and reliability of the Multidimensional Scale of Perceived Social Support. Turk. Psikol. Derg..

[24] Ekşi H., Kardaş S. (2017). Spiritual well-being: Scale development and validation. Spirit. Psychol. Couns..

[25] Haydari N., Dejbakhat M., Akbarzadeh M. (2022). A study of pregnancy-related anxiety with spiritual health among cesarean and vaginal delivery. Curr. Womens Health Rev..

[26] Polat F., Karasu F., Yıldız M. (2022). The effect of religious attitudes on anxiety and psychological well-being in risky pregnancies: A cross-sectional study from Turkey. J. Relig. Health.

[27] Bakir N., Irmak Vural P., Demir C. (2021). Relationship of depression, anxiety and stress levels with religious coping strategies among Turkish pregnant women during the COVID-19 pandemic. J. Relig. Health.

[28] Piccinini C.R.P., de Castro Almeida V., da Silva Ezequiel O., de Matos Fajardo E.F., Lucchetti A.L.G., Lucchetti G. (2021). Religiosity/spirituality and mental health and quality of life of early pregnant women. J. Relig. Health.

[29] Battulga B., Benjamin M.R., Chen H., Bat-Enkh E. (2021). The impact of social support and pregnancy on subjective well-being: A systematic review. Front. Psychol..

[30] Chauhan A., Potdar J. (2022). Maternal mental health during pregnancy: A critical review. Cureus.

[31] Grzesik-Gąsior J., Zalewska K., Pieczykolan A., Kowalski S., Żak-Kowalska K., Niewiadomska I., Bień A. (2025). The mediating role of social support between pregnancy anxiety and emotional suppression in women with threatened preterm labor. J. Clin. Med..

